# The intersectional effects of ethnicity/race and poverty on health among community-dwelling older adults within multi-ethnic Asian populace: a population-based study

**DOI:** 10.1186/s12877-021-02475-5

**Published:** 2021-09-27

**Authors:** Hui Foh Foong, Tengku Aizan Hamid, Rahimah Ibrahim, Sharifah Azizah Haron

**Affiliations:** 1 Malaysian Research Institute on Ageing (MyAgeingTM), Universiti Putra Malaysia, 43400 Serdang, Selangor Malaysia; 2 Department of Human Development and Family Studies, Faculty of Human Ecology, Universiti Putra Malaysia, 43400 Serdang, Selangor Malaysia; 3 Department of Resource Management and Consumer Studies, Faculty of Human Ecology, Universiti Putra Malaysia, 43400 Serdang, Selangor Malaysia

**Keywords:** Ethnicity/race, Health differences, Mental health, Physical health, Poverty

## Abstract

**Background:**

Ethnicity/race and poverty are among determinants of health in older persons. However, studies involving intersectional effects of ethnicity/race and poverty on health of older adults within multi-ethnic Asian populace is limited. Hence, this study aimed to examine the intersectional effects of ethnicity/race and poverty on cognitive function, depressive symptoms, and multimorbidity among community-dwelling older adults in Malaysia.

**Methods:**

Data were obtained from the first wave of a Peninsular Malaysia national survey - “Identifying Psychosocial and Identifying Economic Risk Factor of Cognitive Impairment among Elderly”. Log-binomial regression was used to identify intersectional effects and associations between control variables and health outcomes. A comparison between Malay and non-Malay older adults within the same poverty group, as well as hardcore poor and non-hardcore poor older people within the same ethnicity groups, were conducted to understand the intersectional effects of ethnicity/race and poverty on health.

**Results:**

Prevalence of cognitive impairment was highest among hardcore poor Malay group, while the risk of depression and multimorbidity were highest among hardcore poor non-Malays. In the hardcore poor group, Malay ethnicity was associated with higher prevalence of cognitive impairment but lower prevalence of depression risk and multimorbidity. In the Malay group, hardcore poor was associated with higher prevalence of cognitive impairment; however, no association was found between hardcore poor with depression risk and multimorbidity after controlling for covariates.

**Conclusions:**

Health outcomes of Malaysian older adults differ according to ethnicity and socioeconomic status. Given the importance of demographic characteristics on health outcomes, design of interventions targeting older adults within multi-ethnic settings must address specific characteristics, especially that of ethnicity and sociodemographic status so as to fulfil their needs. Several implications for future practice were discussed.

## Introduction

Contemporary discussion within the field of geriatrics lies significantly on the topic of health disparities among older adults. Health disparities is defined as differences in health outcome due to economic, social or environmental disadvantages [1]. Problems associated with health disparity includes but is not limited to delayed healthcare utilisation, poor health status, and reduced cognitive vitality among older adults [2]. Studies have reported ethnic diversity and poverty as undeniable causes of health disparities among older adults [2, 3].

### Poverty, ethnicity, and health outcome

Past studies deduces an association between old age poverty with disabilities, such as physical, mental, and sensory disability, especially in low- and medium-income countries [4]. In terms of mental health disparities, poverty has been closely associated with cognitive impairment and depression. For example, a study has shown that higher current poverty index score in older adults was independently associated with poorer cognitive function, despite adjusting for educational attainment [5]. Furthermore, a nine-year longitudinal study also showed that older adults of low socioeconomic status had higher incidences of depression [6].

Old age poverty is also linked to multimorbidity [7]. A recent study from Brazil revealed older adults living in poverty had shorter life expectancy and higher prevalence of multimorbidity [8]. The association between poverty and health could be attributed to several factors, including material conditions (e.g., food availability, healthcare utilisation), lifestyle practices, discriminatory practices, work conditions, community conditions, and government policies [9].

Levels of cognitive function was shown to differ ethnically. Diaz-Venegas et al. reported that older adults in the United States whom were Hispanics and non-Hispanic blacks had lower levels of cognitive function compared to non-Hispanic whites [10]. Though cognitive functions differ according to ethnicity, education remains to be the most potent determinant of old age cognitive function [10, 11]. Studies also unveiled differences in prevalence of depression among older adults according to race and ethnicity. For example, prevalence of depression was higher among Mexicans, Puerto Ricans, Cubans, including other Hispanics or Latinos, compared to non-Hispanic whites [12]. These differences might be due to differing perceptions of need for mental health service among ethnicities[13]. Big data analysis of electronic medical records affirms that physical health may differ ethnically [14]. According to St Sauver et al., incidence rates of multimorbidity drastically increased after 65 years old. Incidence of multimorbidity were higher among blacks and lower in Asian Americans in comparison to whites [15].

### Ethnicity and poverty distribution of Malaysian older adults as well as healthcare system in Malaysia

Malaysia is a multi-ethnic nation. The population tally for 2019 consisted of 29.4 million citizens, with the ethnic majority being *Bumiputera* (69.3 %), followed by Chinese (22.8 %), Indians (6.9 %), and other (1.0 %) ethnic groups [16]. *Bumiputera* in Peninsular Malaysia refers to Malays and indigenous peoples of Southeast Asia [17]. Where ethnicity and socioeconomic status among older adults were concern, a large number of Malays and indigenous people resided in rural areas and were more socioeconomically disadvantaged compared to ethnic Chinese and Indians [18]. Most older Malays also had minimal or no education, were involved in traditional agriculture and earned lower compared to other ethnic groups. The same disadvantages applied to older women, whom rarely worked and had no other source of income, other than private transfers from their children [18, 19].

Healthcare services in Malaysia provides equal treatment to all and it can be divided into two facets - government and private. Cost of treatment for Malaysians at government healthcare facilities is considerably affordable as due to heavy subsidisation by the government [20]. In fact, the share of health expenditure in Malaysia’s GDP for 2017 was 3.9 % [21]. Most could afford government healthcare facilities, even those within the hardcore poor category, as registration costed only RM 1 (USD 0.25) for outpatient treatment and RM 5 (USD 1.24) for specialist treatment. Malaysian healthcare accessibility is also commendable as majority rural areas were equipped with community clinics and district hospitals. Emergency or referral cases from rural areas are usually referred to the nearest hospital. Private hospitals and general practitioners on the other hand are easily available in Malaysian urban vicinities. Cost of services at private health facilities are higher compared to government facilities but its patrons deem private facilities to have shorter waiting time and more comfortable ward environment.

### Justification, theoretical perspective, and objective of the study

Information relevant to the intersectional effects of ethnicity and poverty on health in a multi-ethnic Asian nation remain limited. The gap is one that requires crucial exploration as findings from multi-ethnic populations could help identify disadvantaged group associated towards a specific health condition, besides understanding differing health needs of ethnic groups. Findings obtained could be used to determine whether there is a need for improvement of current healthcare system or development of community-based healthcare facility, in line with achieving principle two of the Sustainable Development Goals - Leave No One Behind. The present study was guided by the intersectionality theory that posits that multiple social categories such as race, ethnicity, sex, and socioeconomic status intersect at the micro-level of individual experience to reflect multiple interlocking systems of privilege and oppression at the macro, social-structural level [22]. More recently, scholars began using intersectional approaches to examine the complex configurations of social determinants of health and how those social constructs interact to cause health disparities [23]. In this study, we viewed ethnicity/race and socioeconomic status as important social determinants of health. We would like to investigate whether their intersection could link to health disparities in older adults living in a multi-ethnicity Asia country. Therefore, the primary aim of this study was to identify the intersectional effect of ethnicity/race and poverty on health status among community-dwelling older Malaysian adults by examining disparities in cognitive function, depression status and multimorbidity. Given the general scarcity of studies relating intersectional effects of poverty and ethnicity on health in a multi-ethnic elder population, hypotheses were developed based on the fact that Malay older adults were more deprived socioeconomically compared to non-Malay in Malaysia. Hypotheses postulated were intersecting hardcore poor and Malay ethnicity would fare worse for cognitive impairment (H_1_), at risk of depression (H_2_), and multimorbidity (H_3_) compared to others.

## Methods

### Data source

Baseline data of the longitudinal study, “Identifying Psychosocial and Identifying Economic Risk Factor of Cognitive Impairment among Elderly” were used. Data were collected from more than 2000 community-dwelling older adults in 2015 via population-based health survey of four states within Peninsular Malaysia. The study collected 2322 responses from community-dwelling older adults with 90 % of response rate. Multistage, proportional cluster, random sampling technique was used, involving face-to-face interview of one participant per household. Proportions of varying age groups, sex, locality (urban VS rural), and ethnicities of chosen sample was representative of community-dwelling older adults residing in Peninsular Malaysia. Detailed methodology of this study has been published separately [24].

### Measures

*Ethnicity* was measured through the question “What is your race?”. Answer options provided were Malay, Chinese, Indian, and others but these options were further dichotomised to Malay and non-Malay (Chinese, Indian, others).

*Poverty status* was determined via self-reported household income measures. Household income was defined as combined gross income of all occupants aged 15 and above within the same housing unit. Individuals classified under the hardcore poverty group were identified with reference to the Poverty Line Income (PLI). The PLI measures the household ability to meet minimum requirement of food and non-food consumption. For this study, the 2013 PLI level cut-off point was used, where households in Peninsular Malaysia with income less than MYR 460 (approximately USD 113) were considered hardcore poor [25].

*Depressive symptoms* were assessed via Geriatric Depression Scale-15 [26]. The scale consists of 15 yes/no items, with score ranging from 0 to 15. Scores of 4 and above were indicative of “at risk of depression” [26].

*Cognitive function* was measured via Malay language Montreal Cognitive Assessment [27]. Based on Che Din et al., score of 17/18 was a suitable cut-off point to detect mild cognitive impairment detection among Malaysian older adults with 68.2 % sensitivity and 61.3 % specificity [28]. Therefore, participants with scores 18 and above were categorised as no cognitive impairment, whereas participants with a score of less than 18 were labelled as cognitively impaired.

*Multimorbidity* was measured based on self-reported medical history. Respondents were asked, “Has healthcare practitioner ever told you that you had … that require ongoing medical attention and/or limit activities of daily living?” The list included twelve common geriatrics diseases. Older adults with two or more chronic diseases were categorised under multimorbidity. The complete list of diseases and studies utilising similar method to assess multimorbidity have been presented separately [29, 30].

*Covariates* included were sex (men vs. women), age (60-70-year vs. > 71-year), marital status (married vs. non-married), education level (no formal education vs. primary education and higher), occupational status (currently working vs. currently not working) and living arrangement (living alone vs. living with others).

### Analytical plan

The original dataset consisted of 2322 respondents. However, current analyses involved respondents with household income information, resulting in a sample of 2196 (94.6 % of the original dataset) with data on poverty status and ethnicity. No missing data was found for ethnicity. In the sample with 2196 responses, there were missing data for employment status (0.8 %), cognitive function (1.8 %), and depression status (1.6 %). As the percentage of missing data was less than 10 % and the pattern of missing data was missing completely at random (MCAR) (Little’s MCAR test: chi-square = 2.447, *df* = 5, *P* = 0.784), pairwise deletion was used to handle the missing data without biased [31]. As showed in Table 1, this study involved four comparison groups - non-hardcore poor Malay (*n* = 877), hardcore poor Malay (*n* = 503), non-hardcore poor non-Malay (*n* = 521), and hardcore poor non-Malay (*n* = 295). Univariate analysis (i.e., frequency and percentage) was used to explore sample characteristics and prevalence of cognitive impairment, at risk of depression, and multimorbidity among the four aforementioned comparison groups were identified. Bivariate analysis (i.e., chi-square statistic) was used to examine associations between poverty status and sex, age, marital status, education level, employment status, and living arrangement of Malay and non-Malay groups. Since the binary outcomes were common (prevalence rates above 10 %), log-binomial regression was used to examine the influence of intersecting Malay ethnicity and poverty status on cognitive impairment, depression status, and multimorbidity by estimating the prevalence ratio (PR). PR should be used instead of odds ratio (OR) in cross-sectional studies because the PR can be overestimated by the OR when the PR is greater than 1 or underestimated when the PR is less than 1 [32]. In this study, PR was estimated by running log-binomial regression, a generalised linear model with a binomial distribution and logarithmic link function [33]. The problem of convergence is a common issue in log-binomial regression, especially with continuous independent variables [34]. In the current analysis, we included all covariates as categorical variables; therefore, convergence was not a concern.

**Table 1:** Demographic characteristics of the sample by poverty status and ethnicity

Demographic characteristics	Malay (*n* = 1380)		Non-Malay (*n* = 816)		Chi-square	*P*-value
	Non-hardcore poor (*n* = 877)	Hardcore poor (*n* = 503)	Non-hardcore poor (*n* = 521)	Hardcore poor (*n* = 295)		
	*n* (%)	*n* (%)	*n* (%)	*n* (%)		
**Sex**						
Men	478 (54.5)	213 (42.3)	254 (48.8)	96 (32.5)	49.406	**< 0.001**
Women	399 (45.5)	290 (57.7)	267 (51.2)	199 (67.5)		
**Age**						
60–70 years old	618 (70.5)	274 (54.5)	339 (65.1)	149 (50.5)	57.248	**< 0.001**
71 years and above	259 (29.5)	229 (45.5)	182 (34.9)	146 (49.5)		
**Marital status**						
Married	638 (72.7)	274 (54.5)	385 (73.9)	197 (66.8)	59.940	**< 0.001**
Not married (never marry, widowed, divorced, separated)	239 (27.3)	229 (45.5)	136 (26.1)	98 (33.2)		
**Education level**						
Primary education and above	760 (86.7)	342 (68.0)	449 (86.2)	174 (59.0)	152.552	**< 0.001**
No formal education	117 (13.3)	161 (32.0)	72 (13.8)	121 (41.0)		
**Employment status**						
Currently working	256 (29.6)	95 (18.9)	114 (22.1)	30 (10.2)	53.756	**< 0.001**
Currently not working	610 (70.4)	407 (81.1)	401 (77.9)	265 (89.8)		
**Living arrangement**						
Living with others	823 (93.8)	416 (82.7)	465 (89.2)	254 (86.1)	44.457	**< 0.001**
Living alone	54 (6.2)	87 (17.3)	56 (10.7)	41 (13.9)		

## Results

### Background characteristics of the sample by poverty status and ethnicity

Table 1 depicts demographic characteristics of sample according to poverty status and ethnicity. The study involved 1380 Malay respondents and 816 non-Malay respondents. Non-Malay sample consisted of Chinese (*n* = 708) and Indian (*n* = 108) ethnicity. Prevalence of hardcore poor among Malays and non-Malays were 36.4 % and 36.2 % respectively. Detailed demographic characteristics of the sample in use is available separately [35].

Gender distribution of men (*n* = 691, 50.1 %) and women (*n* = 689, 49.9 %) among the Malay ethnic group was almost equal. Most of the respondents within the Malay group were within the age group 60–70 years old (*n* = 892, 64.6 %), were married (*n* = 912, 66.1 %), received at least primary school education (*n* = 1102, 79.9 %), currently not working (*n* = 1017, 74.3 %) and were living with others (*n* = 1239, 89.8 %). As observed in Table 1, chi-square analysis found significant associations (*P* < 0.001) between sex, age, marital status, education level, employment status, living arrangement, with poverty status among Malays and non-Malays. It was also observed that hardcore poor were observed mostly among women, individuals aged 60–70, married individuals, those with primary education and above, those currently not working, and those living with others. The trends were similar across Malay and non-Malay groups.

### The prevalence of cognitive impairment, at risk of depression, and multimorbidity

Table 2 depicts the prevalence of cognitive impairment, risk of depression, and multimorbidity within overall sample, hardcore poor Malays, non-hardcore poor Malays, hardcore poor non-Malays, and non-hardcore poor non-Malays, respectively. Prevalence of cognitive impairment, risk of depression, and multimorbidity among overall sample were 45.2 %, 16.5 %, and 50.4 %, respectively. Hardcore poor Malays had highest prevalence of cognitive impairment (64.2 %), but hardcore poor non-Malays were highly at risk of depression (29.0 %) and had highest prevalence of multimorbidity (57.6 %).

**Table 2:** The prevalence of cognitive impairment, at risk of depression, and multimorbidity among older adults in overall sample, hardcore poor Malay, non-hardcore poor Malay, hardcore poor non-Malay, and non-hardcore poor non-Malay

	Cognitive impairment	At risk of depression	Multimorbidity
	Prevalence		
Overall	45.2	16.5	50.4
Hardcore poor Malay	64.2	16.3	46.5
Non-hardcore poor Malay	39.9	12.2	49.3
Hardcore poor non-Malay	45.0	29.0	57.6
Non-hardcore poor non-Malay	38.6	17.2	51.6

### The associations between poverty status, ethnicity, and cognitive function

Table 3 shows the associations between poverty status and ethnicity on cognitive function. Prior to controlling for covariates, it was found that within the hardcore poor strata, the prevalence of cognitive impairment was 1.425 times greater in Malays than in non-Malays (prevalence ratio [PR] = 1.425, *P* < 0.001). The relationship remained significant (PR = 1.496, *P* < 0.001) even after controlling for covariates. However, no association was found between ethnicity and cognitive function (for both adjusted and non-adjusted model) among non-hardcore poor older adults. There were more older adults living in hardcore poor whom had cognitive impairment in both unadjusted (PR = 1.609, *P* < 0.001) and adjusted model (aPR = 1.383, *P* < 0.001) among the Malays. Upon controlling for covariates, it was found that the prevalence of cognitive impairment in hardcore poor Malay older adults were 1.383 times greater than the non-hardcore poor. In contrast, no association was found between poverty status and cognitive function among non-Malay older adults (see Table 3).

**Table 3:** Test of poverty status and ethnicity on cognitive function

Mental health outcome 1 - Cognitive impairment (ref: no cognitive impairment)	Test of hardcore poverty status^a^				Test of ethnicity^b^			
	Among hardcore poor		Among non-hardcore poor		Among Malay		Among non-Malay	
	PR^c^	95 % CI	PR^c^	95 % CI	PR^c^	95 % CI	PR^c^	95 % CI
Unadjusted^d^ - Malay or hardcore poor (ref: non-Malay in test of hardcore poverty status and non-hardcore poor in test of ethnicity)	1.425***	1.235–2.645	1.035	0.902–1.186	1.609***	1.448–1.787	1.168	0.987–1.381
Adjusted^e^ - Malay or hardcore poor (ref: non-Malay in test of hardcore poverty status and non-hardcore poor in test of ethnicity)	1.496***	1.304–1.716	1.070	0.939–1.220	1.383***	1.249–1.532	1.015	0.849–1.212
Covariates								
Women (ref: men)	0.984	0.858–1.129	1.162	0.993–1.361	1.063	0.963–1.172	1.067	0.875–1.302
71 years old and above (ref: 60–70 years old)	1.132*	1.005–1.275	1.124	0.978–1.292	1.188**	1.075–1.313	1.070	0.902–1.268
Not married (ref: married)	0.966	0.849 -1.100	1.125	0.956–1.323	1.098	0.989–1.220	0.976	0.797–1.195
No formal education (ref: primary education and above)	1.495***	1.311–1.704	1.552***	1.328–1.815	1.514***	1.360–1.686	1.499***	1.229–1.827
Currently not working (ref: currently working)	1.033	0.876–1.218	0.907	0.781–1.054	1.016	0.900-1.147	0.887	0.719–1.093
Living alone (ref: living with others)	1.108	0.988–1.243	1.093	0.893–1.337	0.957	0.860–1.064	1.260	1.002–1.586

Note: ^a^, ethnicity as the independent variable in the test of hardcore poverty status; ^b^, poverty status as the independent variable in the test of ethnicity; ^c^, PR > 1 indicate that the prevalence of cognitive impairment is higher in those with exposure; ^d^, regression of ethnicity or poverty status (ref = non-Malay in test of hardcore poverty status and non-hardcore poor in test of ethnicity) on cognitive function (ref = no cognitive impairment) without covariates; ^e^; regression of ethnicity or poverty status (ref = non-Malay in test of hardcore poverty status and non-hardcore poor in test of ethnicity) on cognitive function (ref = no cognitive impairment) with covariates (ref = men, 60–70 years old, married, primary education and above, currently working, and living with others); PR, prevalence ratio; CI, confidence interval; * *P* < 0.05, ** *P* < 0.01, *** *P* < 0.001

### The associations between poverty status, ethnicity, and depression status

Table 4 reveals association between poverty status and ethnicity on depression status. Where hardcore poor is concerned, there were fewer Malay older adults whom were at risk of depression, for both in unadjusted (PR = 0.564, *P* < 0.001) and adjusted model (aPR = 0.630, *P* = 0.001). As a matter of fact, the prevalence of at risk of depression was 37.0 % lesser in hardcore poor Malay older adults than in hardcore poor non-Malay older adults upon controlling for covariates. Similar pattern was also found among non-hardcore poor, where Malay older adults less likely to be at risk of depression (PR = 0.707, *P* = 0.009; aPR = 0.682, *P* = 0.004). Meaning the prevalence of at risk of depression in non-hardcore poor Malay older adults was 31.8 % lesser compared to hardcore poor non-Malay older adults. Hardcore poverty was found to be associated with depression status (PR = 1.342, *P* = 0.031) among Malays. However, the relationship became non-significant upon controlling for covariates (aPR = 1.171, *P* = 0.264). An association was also observed between ethnicity and depression status among non-Malays. More hardcore poor older adults were at risk of depression (PR = 1.682, *P* < 0.001; aPR = 1.428, *P* = 0.016) among the non-Malays. In specific, hardcore poor non-Malay older adults had a prevalence of depression risk that was 1.682 times greater than non-hardcore poor non-Malays.

**Table 4:** Test of poverty status and ethnicity on depression status

Mental health outcome 2 - At risk of depression (ref: no risk of depression)	Test of hardcore poverty status^a^				Test of ethnicity^b^			
	Among hardcore poor		Among non-hardcore poor		Among Malay		Among non-Malay	
	PR^c^	95 % CI	PR^c^	95 % CI	PR^c^	95 % CI	PR^c^	95 % CI
Unadjusted^d^ - Malay or hardcore poor (ref: non-Malay in test of hardcore poverty status and non-hardcore poor in test of ethnicity)	0.564***	0.431–0.738	0.707**	0.544–0.917	1.342*	1.027–1.755	1.682***	1.294–2.186
Adjusted^e^ - Malay or hardcore poor (ref: non-Malay in test of hardcore poverty status and non-hardcore poor in test of ethnicity)	0.630**	0.477–0.832	0.682**	0.525–0.887	1.171	0.883–1.552	1.428*	1.069–1.908
Covariates								
Women (ref: men)	1.063	0.771–1.465	0.975	0.712–1.335	0.982	0.709–1360	1.110	0.811–1.519
71 years old and above (ref: 60–70 years old)	0.983	0.743-1.300	1.153	0.868–1.530	1.045	0.777–1.406	1.065	0.807–1.405
Not married (ref: married)	0.676*	0.481–0.951	1.186	0.853–1.651	0.835	0.584–1.194	0.928	0.670–1.285
No formal education (ref: primary education and above)	1.640**	1.227–2.193	2.147***	1.572–2.932	2.269***	1.664–3.095	1.509**	1.107–2.059
Currently not working (ref: currently working)	0.999	0.679–1.471	0.927	0.680–1.265	0.993	0.718–1.372	0.977	0.679–1.404
Living alone (ref: living with others)	1.472*	1.020–2.124	0.641	0.363–1.130	0.986	0.619–1.572	1.137	0.760–1.701

Note: ^a^, ethnicity as the independent variable in the test of hardcore poverty status; ^b^, poverty status as the independent variable in the test of ethnicity; ^c^, PR > 1 indicate that the prevalence of at risk of depression is higher in those with exposure; ^d^, regression of ethnicity or poverty status (ref = non-Malay in test of hardcore poverty status and non-hardcore poor in test of ethnicity) on depression status (ref = no risk of depression) without covariates; ^e^; regression of ethnicity or poverty status (ref = non-Malay in test of hardcore poverty status and non-hardcore poor in test of ethnicity) on depression status (ref = no risk of depression) with covariates (ref = men, 60–70 years old, married, primary education and above, currently working, and living with others); PR, prevalence ratio; CI, confidence interval; * *P* < 0.05, ** *P* < 0.01, *** *P* < 0.001

### The associations between poverty status, ethnicity, and multimorbidity

Table 5 depicts association between poverty status and ethnicity on multimorbidity. Prevalence of multimorbidity was lesser among hardcore poor Malay older adults compared to hardcore poor non-Malay individuals (PR = 0.807, *P* = 0.002; aPR = 0.805, *P* = 0.002). Based on the adjusted model of hardcore poor adults, Malays reported 19.5 % lesser prevalence of multimorbidity compared to non-Malays. On the contrary, no association was found between ethnicity and multimorbidity among non-hardcore poor older adults. No association was also found between poverty and multimorbidity in both Malays and non-Malays (see Table 5).

**Table 5:** Test of poverty status and ethnicity on multimorbidity

Physical health outcome - multimorbidity (ref: no multimorbidity)	Test of hardcore poverty status^a^				Test of ethnicity^b^			
	Among hardcore poor		Among non-hardcore poor		Among Malay		Among non-Malay	
	PR^c^	95 % CI	PR^c^	95 % CI	PR^c^	95 % CI	PR^c^	95 % CI
Unadjusted^d^ - Malay or hardcore poor (ref: non-Malay in test of hardcore poverty status and non-hardcore poor in test of ethnicity)	0.807**	0.705–0.924	0.954	0.857–1.062	0.944	0.842–1.060	1.274	0.955–1.699
Adjusted^e^ - Malay or hardcore poor (ref: non-Malay in test of hardcore poverty status and non-hardcore poor in test of ethnicity)	0.805**	0.701–0.925	0.981	0.882–1.091	0.955	0.850–1.073	1.041	0.906–1.195
Covariates								
Women (ref: men)	0.935	0.797–1.095	1.017	0.905–1.143	0.983	0.869–1.113	0.980	0.846–1.137
71 years old and above (ref: 60–70 years old)	0.905	0.783–1.046	1.069	0.954–1.198	0.999	0.883–1.129	1.041	0.910–1.191
Not married (ref: married)	1.091	0.928–1.282	0.970	0.844–1.114	0.944	0.820–1.087	1.149	0.985–1.339
No formal education (ref: primary education and above)	0.957	0.820–1.117	0.841	0.705–1.004	0.790**	0.666–0.937	1.030	0.880–1.205
Currently not working (ref: currently working)	1.268*	1.010–1.592	1.446***	1.248–1.675	1.501***	1.283–1.756	1.250*	1.025–1.524
Living alone (ref: living with others)	1.096	0.907–1.325	1.014	0.822–1.251	1.002	0.820–1.226	1.075	0.889–1.299

Note: ^a^, ethnicity as the independent variable in the test of hardcore poverty status; ^b^, poverty status as the independent variable in the test of ethnicity; ^c^, PR > 1 indicate that the prevalence of multimorbidity is higher in those with exposure; ^d^, regression of ethnicity or poverty status (ref = non-Malay in test of hardcore poverty status and non-hardcore poor in test of ethnicity) on multimorbidity (ref = no multimorbidity) without covariates; ^e^; regression of ethnicity or poverty status (ref = non-Malay in test of hardcore poverty status and non-hardcore poor in test of ethnicity) on multimorbidity (ref = no multimorbidity) with covariates (ref = men, 60–70 years old, married, primary education and above, currently working, and living with others) ; PR, prevalence ratio; CI, confidence interval; * *P* < 0.05, ** *P* < 0.01, *** *P* < 0.001

## Discussion

This study aimed to examine the intersectional effects of ethnicity and poverty on mental and physical health among Malaysian older adults. Hypotheses of the present study were the prevalence of cognitive impairment (H_1_), at risk of depression (H_2_), and multimorbidity (H_3_) would be higher among those intersecting hardcore poor and Malay ethnicity. H_1_ is supported, evidenced by the association between Malay ethnicity and cognitive impairment in the hardcore poor group and the presence of a significant relationship between hardcore poor and cognitive impairment in the Malay group. The findings indicated that those intersecting hardcore poor and Malay ethnicity had a higher tendency for cognitive impairment. This finding is consistent with previously published Singaporean study, where Malays were almost twice more likely to have cognitive impairment compared to other ethnicities despite controlling for demographic characteristics and cardiovascular health status [36]. Another population-based study in Malaysia also found that dementia in older adults were highly prevalent among the Malays [37]. The underlying reason for the association between ethnic Malay and cognitive impairment remains unknown as further analysis of the same dataset showed no significant difference in years of education between Malays and non-Malays. However, there could be a cultural factor at play. Malays tend to view cognitive impairment among older adults as part of the normal ageing process and seeking mental health treatment were often disregarded as unnecessary. There was a general stigma that existed within the community when seeking mental health treatment in healthcare facilities. All of which could have created a barrier for older patients seeking treatment in healthcare facilities [38]. Past studies also suggest old age poverty is strongly linked with cognitive impairment. Older adults living in poverty did not have the resources required to maintain high cognitive function, like access to healthcare facilities and nutritional benefits [39, 40]. Some researchers argue poverty itself is a cause for loss of cognitive capacity as poverty-related concerns may consume available mental resources, leaving less for tasks (30).

The second hypothesis, H_2_ postulated by the study, however, were not supported when findings obtained revealed that Malays had lower prevalence of depression risk despite their hardcore poor status. Contrarily, hardcore poor non-Malay older adults had highest prevalence of depression risk. Risk of depression in general were more prevalent among non-Malays. These findings appear inconsistent with a Singaporean study that discovered that Malays and Indians, both of which are minority ethnics within the nation, were more prone to depression compared to Chinese [41]. A cross-sectional study conducted in a northern state of Peninsular Malaysia found that prevalence of depression among older adults living in poverty was 19.2 %. In fact, those whom were poor were 2.7 times likely to have severe depression compared to those who were not poor (38). The low prevalence of depression risk among Malays could be attributed to their religious practices. Malays in Malaysia are Muslims that firmly adhere to the Islamic spiritual practice. An important value brought forth from old Muslim practices is “*redha*”, which means to wholeheartedly accept the decree of the Almighty. As a result, Malay older adults are generally happy and content with their lives though most live in poverty. This is validated through further analysis of the same dataset that reported higher intrinsic and extrinsic religiosity scores among Malay older adults compared to non-Malay older people. Religiosity has been shown by past research to protect against depression and improve well-being among older adults [30, 42].

Findings from the study also did not support H_3_ postulated, when fewer hardcore poor Malay older adults reported multimorbidity, and no association was found between Malay ethnicity and multimorbidity among the non-hardcore poor. Further, prevalence of multimorbidity was highest among hardcore poor non-Malays. These findings indicate that Malay older adults living in poverty were less likely to report multimorbidity compared to non-Malays. This could be attributed to the life expectancy of non-Malays in the current study. In Malaysia, the Chinese have the highest life expectancy at birth [16], and therefore, they tend to report higher numbers of chronic diseases than Malays. Besides, these findings should be interpreted with caution due to the use of self-reported medical history. It is also important to bear in mind that some older Malay may not be aware of existing chronic medical conditions due inconsistent medical check-ups. Prior research claims that among Malaysians, Chinese are more health-conscious and utilise healthcare facilities more than Malay and Indian older adults [43].

The study acknowledges several limitations. Firstly, the use of self-reported measure to identify household income and multimorbidity. Single self-reported measurement of poverty status would cause measurement bias as monthly household income is not the only criteria to assess socioeconomic status. Consequently, the prevalence of hardcore poor may have been over-reported as respondents tend to under-report household income. Therefore, future studies should also assess poverty status by asking about the asset’s ownership, geographical location, and cost of living. Prevalence of multimorbidity may have also been under-reported as respondents tend to under-report existing chronic conditions due to social desirability bias. Hence, future study should consider evaluating prevalence and incidences of multimorbidity across ethnicity via electronic medical record, which are more accurate. Next, this study is a cross-sectional study, disallowing corroboration of causal relationship. Future studies should consider longitudinal study design to examine the exact nature of interaction between changes in socioeconomic status with ethnicity to precisely determine health status. Lastly, this study was only conducted in Peninsular Malaysia. Therefore, results obtained could not be generalised to older adults residing in East Malaysia. Future study should perhaps include older people living in East Malaysia because there is a large population of *Bumiputera* (indigenous group) and poverty rate in East Malaysia higher compared to Peninsular Malaysia.

## Conclusions

These findings may help us to understand the intersectional effects of ethnicity and poverty on health among multi-ethnic community-dwelling older adults in Malaysia. Malay older adults, whom happen to be the majority, were more prone to cognitive impairment regardless of their poverty status. Conversely, non-Malay within the hardcore-poor strata had higher risk for depression and multimorbidity. The implications borne through these findings may be beneficial for future practice. For instance, implementation of ethnicity and poverty specific healthcare interventions. There is a clear need for programs focused on preventing cognitive impairment among Malay older adults irrespective of their poverty status. Likewise, healthcare initiatives that involve screening for cognitive impairment should also include more Malay older adults to delay or prevent dementia. Next, depression prevention program should be targeted towards non-Malay older adults living under poverty. In other words, design of future intervention programs should provide due consideration to specific ethnic-centred characteristics so as to meet their needs. Finally, there is a need to validate the association between ethnicity, poverty and multimorbidity via electronic medical records instead of self-reported measures.

## Data Availability

The datasets used and/or analysed during the current study are available from the corresponding author on reasonable request.
